# Long-term follow-up of patients with large segmental bone defects treated with 3D-printed polycaprolactone/tricalcium phosphate scaffolds

**DOI:** 10.1007/s00068-025-02982-9

**Published:** 2025-11-14

**Authors:** Anna J. L. Lodewijks, Margot M.R. Warin, Lotte C. A. van der Broeck, Maria Gabriella Fois, Carmen Lopez-Iglesias, Taco J. Blokhuis, Martijn van Griensven, Martijn Poeze

**Affiliations:** 1 Department of Trauma Surgery, Maastricht University Medical Centre (MUMC+), P. Debyelaan 25, 6229 HX Maastricht, The Netherlands; 2 Institute of Nutrition and Translational Research in Metabolism (NUTRIM), Maastricht University, Maastricht, The Netherlands; 3 Department cBITE, MERLN Institute for technology-inspired regenerative medicine, Maastricht University, Maastricht, the Netherlands; 4 Department of Instructive Biomaterials Engineering, MERLN Institute for technology-inspired regenerative medicine, Maastricht University, Maastricht, the Netherlands; 5 Department of Biohybrid & Medical Textiles (BioTex), AME–Institute of Applied Medical Engineering, Helmholtz Institute, RWTH Aachen University, 52074 Aachen, Germany; 6 Microscopy CORE Lab, Maastricht University, Maastricht, The Netherlands

**Keywords:** Induced membrane technique, Bone defects, 3D-printed PCL/TCP cages, Long-term outcomes, Posttraumatic segmental defects

## Abstract

**Introduction:**

The Induced Membrane Technique (IMT) combined with autologous bone grafting has proven effective in achieving union in many cases of non-healing bone defects. However, this treatment strategy remainschallenging in very large defects (≥ 10 cm). Although the addition of a 3D-printed scaffold could enhance osteoconductivity and retention of cancellous bone, cells, and bioactive materials in the defect, the long-term outcomes of this innovative approach are not yet known. We therefore reviewed all patients with a very large posttraumatic segmental defect of a long bone treated with the IMT using a 3D-printed scaffold, cancellous bone, cells, and bioactive materials.

**Methods:**

From 2018 to 2024, all consecutive patients with a large segmental defect who received a 3D printed scaffold were identified. All patients were treated with the two-stage IMT procedure. The defects were filled in the second stage procedure with a 3D printed scaffold made from poly-ε-caprolactone and Tricalcium Phosphate (PCL/TCP), autologous bone graft (bone marrow aspirate concentrate and RIA-autograft), and biomaterials(P-15 +/- bioactive glass). In the event of complications, samples from the defect site with the PCL/TCP scaffold were collected for analysis using scanning electron microscopy and histology.

**Results:**

A total of ten patients were treated for a segmental bone defect median of 10.5 cm (IQR 3.25) of the femur (*n* = 3) or tibia (*n* = 7). Follow-up of the patients ranges between one and four years. The median age of the patients is 46 (range 23–62). Complications occurred in seven patients (one femur, six tibia). All patients with complications required additional surgeries. One patient had a local osteomyelitis infection at the defect site with complete integration of the scaffold into the proximal and distal tibia; one required negative pressure wound therapy for a soft tissue infection; and five required a revision of the first stage procedure due to surgical site infection (*n* = 3) or failure of the fracture fixation material (*n* = 2). Remarkably, scanning electron microscopy and histology showed limited formation and incorporation of newly formed bone into the scaffold. After revision surgery, all patients are currently improving, and no amputations have been performed so far.

**Conclusion:**

Reconstructive treatment of large bone defects remains challenging. The short-term follow-up of this treatment strategy showed promising results with progress in clinical and radiological follow-up. However, in this small cohort a lack of bone growth at the defect site was found in some of the cases that were clinically performing well. The use of PCL/TCP scaffolds remains a valuable treatment strategy in very large bone defects for retention and extension of the autologous bone and cells, though improvements in fixation, composition or structural properties could enhance their feasibility.

## Introduction

Very large bone defects have historically been, and continue to be, treated with primary amputation in many cases. However, advances in surgical techniques and biomaterials now enable the reconstruction of defects that exceed the natural regenerative capacity of bone. Reconstruction of a bone defect is challenging, and both primary reconstruction using autograft or allograft as an adjunct, or bone transport are widely advocated treatment methods [1]. While each of these strategies offers distinct advantages and limitations, the ideal treatment has not yet been found [2].

The induced membrane technique (IMT), initially described by Masquelet et al., is a two-stage surgical procedure for treating bone defects [3]. First, the defect is debrided and filled with a cement spacer to induce membrane formation. A second procedure is performed after several weeks, during which the spacer is removed, and the induced membrane is filled with grafting material. The simplicity of the procedure and its favourable success rates have contributed to growing interest in the use of IMT. However, in defects over 6 cm in length, the efficacy of the treatment declines and the need for graft extenders increases since the limited amount of harvested cancellous bone and cells can be insufficient in large defects [4–6]. A key requirement for graft extenders is that they possess osteoconductive or bioactive properties.

Poly-ε-caprolactone (PCL), a biodegradable polymer, is frequently used as a graft extender. When coated with tricalcium phosphate (TCP), the material acquires osteoconductive properties [7, 8]. PCL can be 3D printed to match the dimensions of a patient’s defect [9]. PCL-TCP scaffolds have shown promising effects in osteoconductivity and osteodifferentiation in vitro [10]. Additionally, these scaffolds performed well in various animal models and human case studies [11–14]. The biodegradable properties of the material ensure integration into the newly formed bone over time, which is ought to be around 3 years after implantation [15–17].

The PCL/TCP scaffolds can, within the concept of the induced membrane technique, be used to treat very large defects. In theory, the material provides a bone-friendly environment and supports the containment of other biomaterials, such as cells, autograft, or bioactive peptides. The initial clinical progression of patients with large defects treated with a PCL/TCP scaffold, in combination with an ongoing bone formation on radiographic imaging, created a promising outlook [18, 19]. However, the long-term behaviour of these scaffolds is unknown.

In this retrospective cohort, we present all cases that have been treated with the IMT in combination with a 3D printed PCL/TCP scaffold for a very large posttraumatic bone defect. The aim of this study is to provide a comprehensive overview of the patient population treated with this technique, along with the potential challenges. Additionally, histological and electron microscopic analyses were conducted to gain deeper insight into these challenges and into the in vivo behaviour of the PCL/TCP material.

## Methods

This study was approved by the local ethical committee. Patients were included in this cohort if they met the following criteria: Treatment for a traumatic bone defect using the IMT, addition of a 3D printed PCL/TCP scaffold and a follow-up duration of one year minimum. Patients with a shorter follow-up duration or non-traumatic bone defects were excluded. Data collected for this case series were: Age, gender, fracture type, injury mechanism, surgeries prior to presentation, additional surgeries between the first and second stage IMT, bacterial cultures, grafting materials used during the second stage IMT, complications and clinical progress afterwards. In the event of complications that required revision surgery, samples from the defect site containing the PCL/TCP scaffold were collected for analysis using scanning electron microscopy (SEM) and histology. Radiographs and CT scans were employed to correlate radiological findings with histological and electron microscopic analyses.

### Surgical procedures and grafting materials

All procedures were carried out by a surgical team specialized in non-union surgery of the Maastricht University Medical Center. After initial stabilisation and first stage IMT, the defects were filled during the second stage procedure with the PCL-TCP scaffold, autografts, cells and bioactive materials. The 3D-printed scaffolds (*Osteopore*, Singapore, PCL/TCP, *n* = 9)(TruMatch, *DePuy Synthes*, JJ, United States, PCL alone, *n* = 1).The bioactive graft materials that were used to fill all defects were Bone Marrow Aspirate Concentrate (BMAC) and Reamer-Irrigator-Aspirator derived graft (RIA). For the retrieval of BMAC, a bone marrow puncture of the anterior iliac crest was performed. In short, approximately 100 cc of bone marrow was aspirated and centrifuged to yield between 4 and 6 cc of BMAC (Angel system, *Arthrex*, United States). By reaming of the intramedullary canal of the femur, bone marrow and cancellous bone were retrieved (RIA-2, *Johnson and Johnson*, United States*)* from the ipsilateral femur in case of a tibial defect or from the opposite side in case of a femoral defect. In one case, blood, bone marrow and bone fragments suctioned from the surgical field using a filtering device (BoneFlo, *TissueFlow*, Germany) were used to saturate a TCP sponge (Cerasorb, *Curasan*, Germany), and used as an additional graft extension [20].

In all cases, a form of biological adjunct was used within the concept of polytherapy. rhBMP-2 (Inductos, *Medtronic*, USA) was added in the defect in one case. Peptide-15 (i-Factor, *Cerapedics*, United States) is a short 15-amino acids long peptide. This peptide is present in collagen type 1, where it functions as a binding site for MSCs, leading to their osteogenic differentiation. This peptide is used as a biological adjunct to enhance early osteogenesis [21]. In case of a suspected or demonstrated presence of infection, bioactive glass (S53P4 Bioglass, *Bonalive*, Finland) was added as a local antimicrobial material [22]. This material was placed in the defect, underneath the induced membrane.

### Scaffold design

**Fig. 1:**
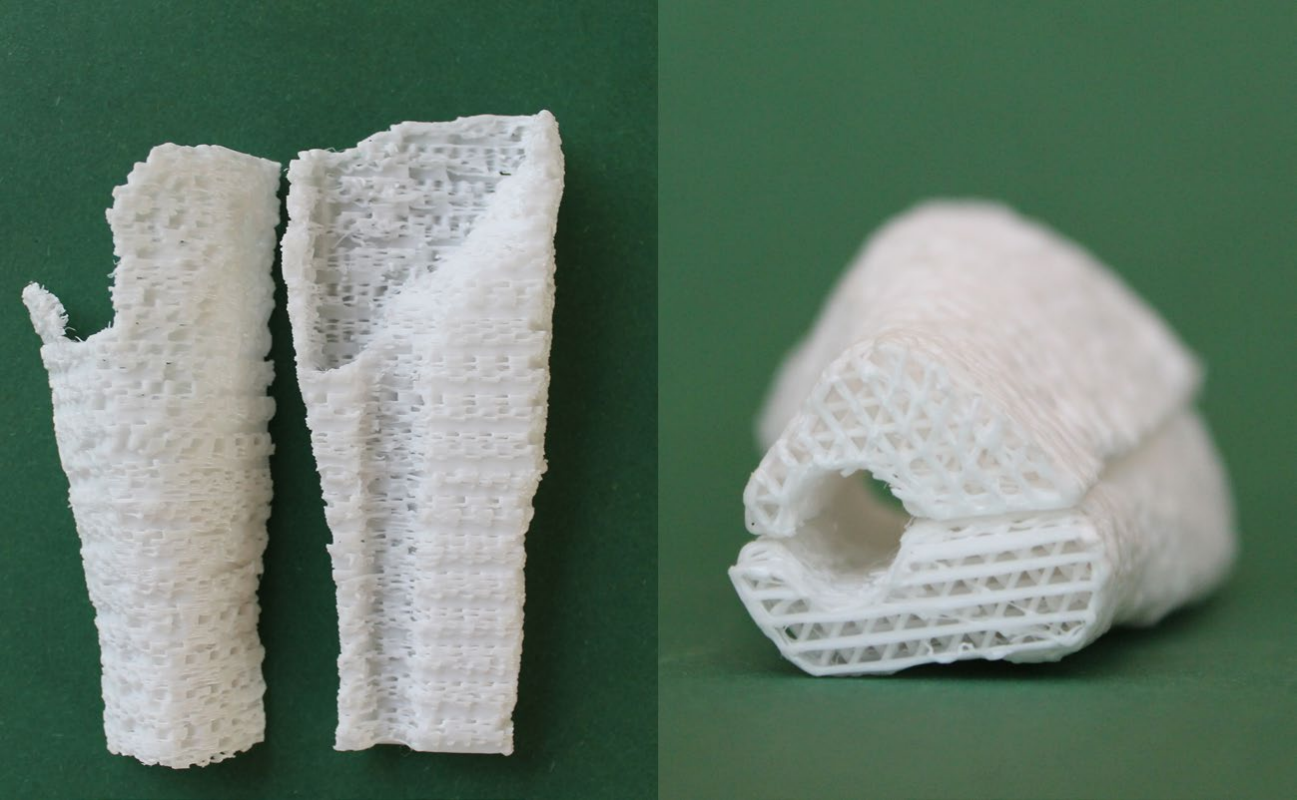
3D printed PCL/TCP scaffold before implantation. This scaffold consists of two parts that can be fitted around an intramedullary nail

All but one of the scaffolds used in this study were manufactured and sterilized by Osteopore (*aXOpore*, Singapore). The scaffolds are modelled based on the CT-scan after the first stage IMT. The Digital Imaging and Communications in Medicine (DICOM) file of the CT-scan is transformed to a Standard Tessellation Language (STL) file, that can subsequently be used to model the 3D-print. By fused filament fabrication, the PCL is printed in a honeycomb structure (0, 60, 120°) that fits the defect. The PCL is coated with TCP, and sterilized by gamma irradiation. The final scaffold consists of 80% PCL and 20% TCP, has a porosity of 70% and a pore size of 3 mm (top-down and sideways). The scaffolds consist of multiple parts to enable fitting in the defect around the fracture fixation material (Fig. 1).

### Follow-up

Patients entered follow-up after their surgical procedure. This follow-up consists of regular visits and follow-up radiographs. Generally, patients will be seen in the outpatient clinic 2, 6, and 12 weeks after surgery and every half year after that. CT scans are performed in case of abnormalities, such as pain or lack of radiological or clinical progression.

### Histology

When patients underwent revision of their defect for any reason, biopsies were obtained. Excised samples were fixed for 24 to 48 h using a 4% paraformaldehyde (PFA, 252549, Sigma-Aldrich) solution. Once fixed, samples were rinsed with Phosphate Buffered Saline (DPBS, D8537, Sigma-Aldrich) and decalcified for 28 days in a 10% ethylenediaminetetraacetic acid solution (EDTA). This 10% EDTA solution was prepared by dissolving EDTA disodium salt (EDTA, E9884, Sigma-Aldrich) in distilled water and adjusting the pH to 7.4 using a 50% sodium hydroxide solution (NaOH, 106462, Merck). The samples were kept under constant agitation using a magnetic stirrer, and a biweekly renewal of the solution was performed. Samples were dehydrated, embedded in paraffin, and cut using a microtome in sections of 7 μm in thickness. After deparaffination, sections were stained using Movat’s Pentachrome (Movat Pentachrome Stain Kit (Modified Russell-Movat), ab245884, Abcam), dehydrated, mounted using Pertex mounting medium (PERTEX^®^, Mounting media, VWR), and imaged with an automated inverted Nikon Ti-E microscope.

### Scanning electron microscopy

Immediately after retrieval of the biopsies, samples containing the scaffold material and adjacent tissue were fixed in a 1.5% Glutaraldehyde (GA)/0.1 ml cacodylate/1% sucrose solution. These were stored at 4 °C until further analyses. Samples were washed with PBS before analysis. To avoid artifacts between the tissue and scaffold material by cutting, samples were analyzed in their entirety. Examination was performed using a scanning electron microscope (JSM IT200, JEOL Ltd.) in low vacuum.

## Results

### Patient treatment and follow-up

Between 2018 and 2024, ten patients were treated using a 3D printed scaffold; 9 males and one female. The median age was 47 years (range 23–62) and the median defect size was 10.5 cm (range 7–15 cm). An overview of all cases can be found in Table 1.

**Table 1:** Overview of the treatment of the ten included patients. * age at time of injury, ** this patient was treated with another scaffold trumatch (Depuy Synthes, JJ, united States)

#	Gender and age*	Fracture type and location (Left/Right)	Defect size (cm)	Trauma mechanism	Events between first and (final) second stage procedure	Second stage procedure				Bacterial cultures after 2nd stage	Complications and procedures after 2nd stage (months after surgery)	Mobility	Successful treatment/Failure
						Autograft	Biological Adjuncts	Osteosynthesis	Soft tissue management				
1	M, 47	Grade 3 A crus (L)	15	Traffic accident	Revision first stage due to infection	RIA BMAC	BMP2	Intramedullary nail	ALT-flap	+	Partial debridement for osteomyelitis (36)	Without aid	Successful
2	M, 45	Grade 3B crus (L)	10	Fall from height	Revision first stage after 2nd stage due to infection	RIA BMAC	iFactor	Plate fixation	ALT-flap	-	Negative pressure therapy for necrosis ALT-flap	Without aid	Successful
3	M, 37	Grade 1 crus (L)	11	Traffic accident	Revision first stage due to wound dehiscence	RIA BMAC	iFactor	Intramedullary nail	ALT-flap	+	Revision malalignment, inert scaffold (38) Revision first stage infection (40) Revision 2nd stage (42)	With aid	Failure
4	F, 62	Grade 3 A crus (L)	13	Crush injury	-	RIA BMAC BoneFlo	iFactor Cerasorb	Intramedullary nail	ALT-flap	-	Revision 1 st stage for nail-break with inert scaffold (25) Revision 2nd stage (30)	With aid	Failure (new scaffold implanted)
5	M, 47	Segmental crus non-union (L)	10	Traffic accident	Revision first stage due to wound dehiscence	RIA BMAC	iFactor Bioglass	Intramedullary nail	ALT-flap	+	Partial resection, nail dynamisation, bioglass for inert scaffold delayed union (20)	With aid	Failure
6	M, 40	Grade 3 A bicondylar distal femur/floating knee	12	Traffic accident	-	RIA BMAC	iFactor	Plate fixation	ALT-flap	-	Revision 1 st stage with inert scaffold and low grade infection (14)	With aid	Failure
7	M, 59	Commuted pilon (R)	8	Fall from height	-	RIA BMAC	iFactor Bioglass	Intramedullary hindfoot nail	ALT-flap	+	Removal nail and partial debridement due to infection (5) Revision 1 st stage with inert scaffold and infection (10)	With aid	Failure
8	M, 59	Grade 3 A crus (L)	7	Traffic accident	Partial flap necrosis	RIA BMAC	iFactor	Intramedullary nail	ALT-flap	-	Removal proximal screw (5)	Without aid	Successful
9	M, 28	Commuted distal femur (L)	11	Traffic accident	-	RIA BMAC	iFactor	Plate fixation	Primary closure	-	Removal lateral plate (9)	With aid	Ongoing/ Successful
10 **	M, 23	Midshaft femur (L)	8	Gunshot	Revision first stage due to infection	RIA BMAC	iFactor Bioglass	Intramedullary nail	Negative pressure therapy	-	-	Without aid	Successful

All patients underwent initial debridement and stabilisation using an external fixator (*n* = 9) or intramedullary nail (*n* = 1) before the first stage of the IMT. The first stage IMT was performed when adequate soft tissue coverage could be achieved by the plastic surgeon. This was achieved by the use of an anterior lateral thigh flap (ALT) in eight cases. In five patients, a revision of the first-stage procedure was needed due to recurrence of infection or failure of the soft tissue coverage before the definitive second stage procedure could be performed. Fixation of the defects was achieved using plate (*n* = 3) or nail fixation (*n* = 7).

The second stage procedure was carried out 17 weeks (range 11–30 weeks) after the first. In all cases, the scaffold was filled with RIA derived autograft prior to implantation. BMAC was combined with the P15-peptide (Flex FR, iFactor, *Cerapedics*, United States) and applied around the scaffold material. In one patient, a TCP sponge (Cerasorb, *Curasan*, Germany) was added as graft extender with material from the surgical site derived using BoneFlo (*TissueFlow*, Germany). Bioactive glass (*Bonalive*, Finland) was used in three patients who had positive bacterial cultures that were obtained in the first debridement. None of these patients displayed clinical signs of infection.

**Fig. 2:**
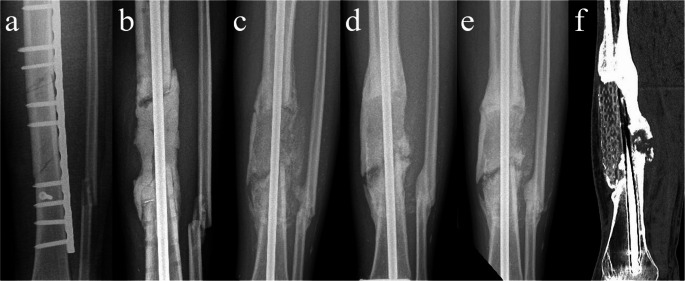
Radiological follow-up of one of the patients (pt.5). Images are in chronological order: **a** – Preoperative image of the initial non-union for which the patient was referred, **b** – Cement in situ after first stage IMT, **c** – After second stage procedure and implantation of the scaffold, **d** – Six months postoperatively with ongoing bone formation proximal and medial, **e** – One and a half year after IMT shows stunted bone formation. The CT-scan (**f**) made after the last radiograph at one and a half year shows a persisting non-union with an inert scaffold

**Fig. 3:**
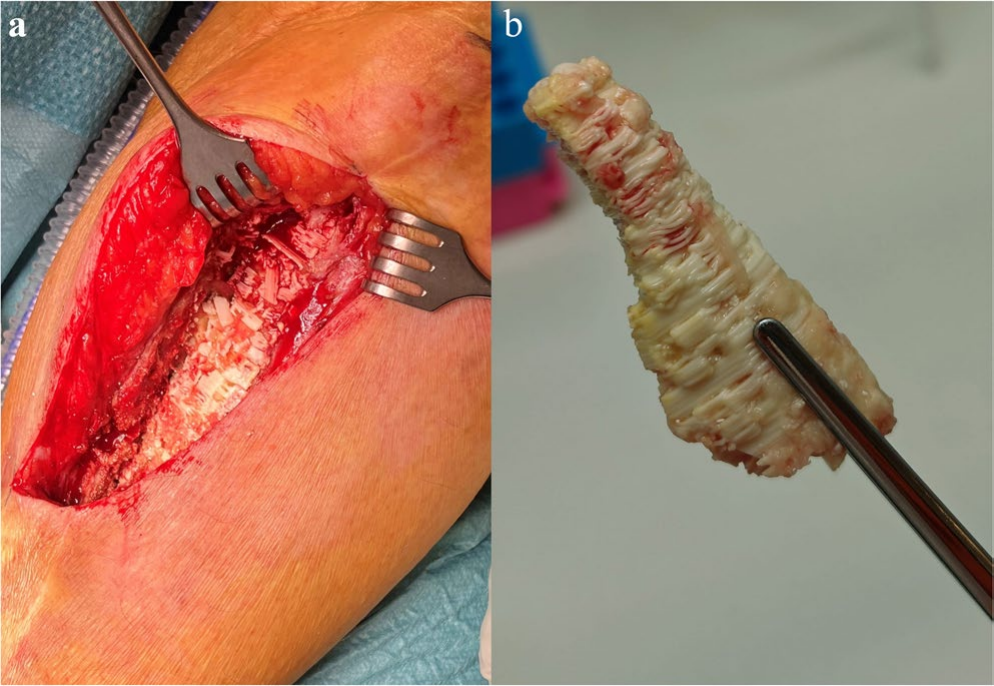
Scaffold in vivo before (**a**, pt.4) and after explantation (**b**, pt.5) for reoperation first stage IMT. Figure 2b. shows one of the scaffold parts that was taken out in its entirety, degradation is visible on the edges and the pores are filled with tissue

Follow-up ranges between 12 and 56 months (median 23 months). The follow-up at six months was uneventful for nine patients, an infection occurred in one. All patients showed increasing bone formation on radiographs and their mobility increased with physical therapy. However, complications occurred in 7 patients (one before 6 months and six after one year). One patient underwent a partial debridement due to a low-grade infection with filling of the defect with bioactive glass (pt.1), and one patient required negative pressure therapy for a wound infection (pt. 2). In both these patients union was achieved after these procedures. Five patients underwent a complete revision of the bone defect with a new IMT procedure. This was due to infection in three out of five patients (pt. 3, 6, 7); One patient experienced a failure of the tibial nail two years after implantation (pt.4) and the last patient showed a persisting non-union on CT after bone formation stunted after one and a half year (pt. 5, Fig. 1e-h). Remarkably, four patients (pt. 3, 4, 5, 6) were pain-free and full weight-bearing without walking aids for over a year (mean 26.8 months) before complications occurred. Since normal radiographic follow-up showed ongoing bone formation in and around the defect, and mobility was improving, these complications were unexpected (Fig. 2a-d). The reoperations were carried out at different times in the follow-up, between 9 and 40 weeks after scaffold implantation, but scaffolds looked the same in vivo regardless of time after implementation. Scaffolds that were removed during the surgery appeared inert with very limited ingrowth of newly formed bone (Fig. 3). Scaffolds that were in situ for a longer period (> 2years) showed more signs of degradation, but again without adequate bone formation.

The initial second stage procedure with a 3D-printed scaffold was successful in five patients, of whom three are discharged from follow-up. Some fracture fixation material was removed due to discomfort (pt. 8, 9), scaffold material of one patient was harvested to investigate the healing process (pt.9). Patients who underwent a complete revision are still in follow-up or awaiting further procedures. All patients are currently weightbearing and radiographic fracture healing is progressing. Some patients returned to sports.

## Histology

**Fig. 4:**
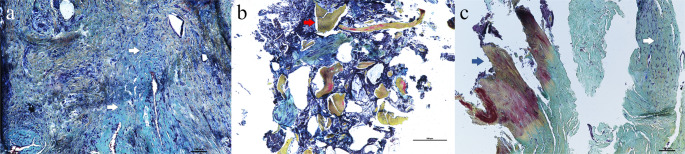
Histology images of patient 3 (**a**, **b**) and 4 (**c**) stained with Movat’s Pentachrome. **a** shows organised connective tissue (white arrows). In **b** the same connective tissue is seen, with non-integrated RIA-derived autograft (red arrow). **c** shows the proximal border of the defect in which the pre-existing mature bone (blue arrow) meets the connective tissue present in the defect (white arrow)

Histology images were obtained from the tissue with the PCL/TCP scaffold from two patients (pt. 3, 4) (Fig. 4). Histologic analysis showed that the samples consist predominantely of connective tissue. No hard callus, newly formed bone tissue, or inflammatory cells were identified. Histology showed isolated laminar mature bone fragments between the connective tissue, which can be identified as fragments from the RIA-derived autograft. The samples of both patients did not show new bone formation.

### Scanning electron microscopy

**Fig. 5:**
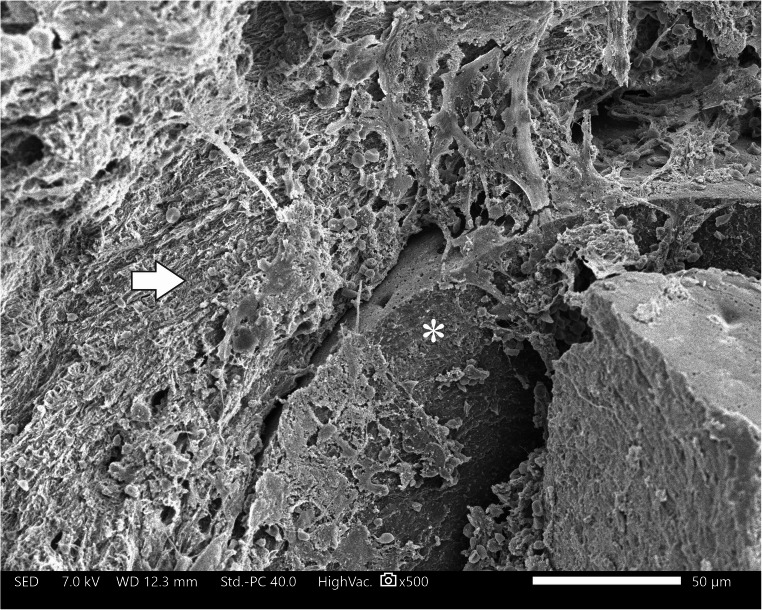
SEM image pt 1. This sample was taken 3 years after the implantation. On the right side, a part of scaffold (*) is seen with mature callus around it at the left side (white arrow). The attachment of the tissue to the scaffold is seen at the top of the scaffold

One patient (pt. 1) healed successfully after partial debridement. SEM showed bridging of the proximal border to the defect site and mature callus surrounding the scaffold material (Fig. 5). After this debridement, the patient did not have any other complications. He returned to full weight bearing and normal activities. The patient refused further follow-up one year later.

**Fig. 6:**
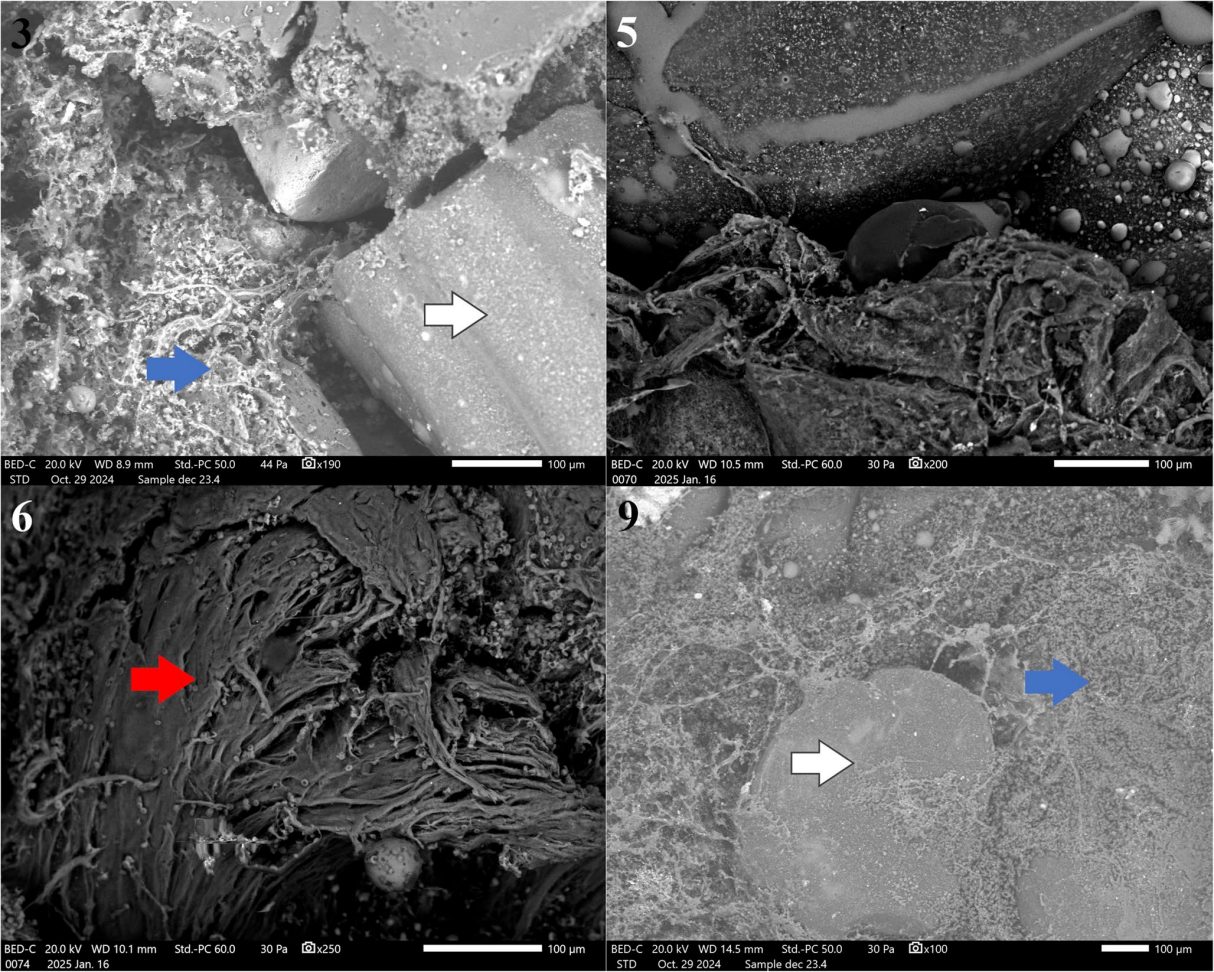
SEM of scaffold samples of patients 3, 5, 6 and 9, taken 40, 20, 14 and 9 months after implantation respectively. The images of patients 3, 5 and 9 show part of the scaffold with connective tissue (blue arrows) surrounding the scaffold (white arrows). Minimal interaction is seen between the tissue and the material. The image of patient 6 shows more organized connective tissue (red arrow)

SEM images of four other patients (3, 5, 6, 9) all showed similar images, with varying stages of connective tissue being formed. The scaffolds were in situ for 40, 20, 14 and 9 months respectively (Fig. 6). The scaffold that was implanted the longest (40 weeks) showed less organised connective tissue and the same amount of degradation of the scaffold material as the other cases. There was no remarkable difference between the two patients with (pt. 3, 6) or without (pt. 5, 9) infection in SEM imaging. All scaffolds were implanted for at least 9 months, during which bone formation was expected to have occurred.

## Discussion

The IMT combined with a 3D-printed scaffold showed to be promising for treating very large bone defects after a short term follow-up [18]. The used scaffold made of PCL-TCP, has previously been shown to support bone formation in (animal) studies [23–26]. However, the long-term results discussed in this study highlight the clear discrepancy between clinical and radiological results in some patients, with a lack of bone growth at the defect site despite radiological and clinical signs of healing. In this series, an unexpected 50% failure rate of the treatment strategy was observed, with failures occurring at a later stage up to three years following the second-stage procedure. To our knowledge, this is the first series describing long-term follow-up including analysis of human biopsies of the PCL/TCP scaffold.

Reoperations of patients were performed for various reasons, including (low grade) infection or failure of fixation. These procedures allowed for the retrieval and analysis of implanted scaffolds or parts thereof using histological examination and SEM. In all cases a similar image was found. The scaffolds were inert, showing absent bone growth in the scaffold and abundant formation of connective tissue. Moreover, the scaffold material showed no sign of resorption even three years after implantation.

Although the findings in this cohort are surprising, high failure rates for critical size defects are not uncommon [27]. Other treatment strategies for large bone defects, such as vascularized autologous bone grafts or bone transport are considered good treatment options. However, vascularized grafts are difficult to perform and result in donor site morbidity [1]. Whereas, the treatment duration of bone transport has a significant impact on the patient’s quality of life [28]. Momentarily, no studies with large cohorts of bone defects over 10 centimetres are published to define a gold standard, and each technique has its own limitations.

This study does have some limitations, mostly because of the study design. This is a retrospective analysis of 10 clinical cases, with a lot of variation in fracture pattern and management. Due to this heterogeneity and the scaffold being only one factor in the complete treatment of these patients, the clinical outcomes might not solely depend on the use of the 3D printed scaffold. However, this cohort is still very valuable, since it does highlight the complexity and heterogeneity of patients suffering from large bone defects. Also, the SEM and histology images are consistent between patients.

The unexpected absence of bone formation in and throughout the scaffold material can be explained by several potential factors. One possible explanation for the lack of bone growth is the presence of a low-grade infection, which was observed in some cases [29]. Three patients who underwent a full revision had positive cultures, which is not surprising, since these patients did have an infection earlier in the treatment process. However, not all patients had positive bacterial cultures. Another explanation is an insufficient osteoinductive stimulus. In one patient who eventually reached union, rhBMP-2 was applied in the defect. Since patients in this cohort did receive different combinations of grafting materials, the union or the lack of union cannot be attributed to a specific material. Evidence of the addition of osteoinductive factors, such as BMPs, in large bone defects is still limited [30]. A third explanation of the lack of bone growth in the scaffold is that the induced membrane itself serves as a physical barrier. This hypothesis is substantiated by the abundance of bone callus that was seen around some of the defects.

Two other possible explanations for the lack of bone healing are related to the scaffold material and the design of the scaffold. First, micro motions are one possible cause for the formation of fibrous tissues, hampering adequate bone formation. Although micro motions are essential to induce fracture healing, too much motion can hinder osteointegration of the graft material and the subsequent bone formation [31, 32]. A review of Kohli et al. found that the ideal amount of movement depends on the site and size of the defect, as well as the biomaterial used to fill it [33]. The scaffolds used in this series are implanted manually in a press-fit manner, but are not fixed to the adjacent bone. Substantial mobility of the scaffolds after implantation therefore, cannot be ruled out. In addition, even if the scaffold material would be fixed to the adjacent bone, the scaffolds consist of multiple parts to facilitate implantation, and these parts are not interconnected. This means that micro motion can cause early and abundant formation of fibrous tissue in the scaffold material, blocking bone ingrowth. A second possible explanation is the structure of the implanted scaffolds. The scaffolds are printed in a honeycomb structure, which creates a scaffold that has a high mechanical strength, as well as a macroporosity that facilitates tissue ingrowth [34, 35]. In vitro studies have shown that scaffold macroporosity significantly influences cell ingrowth and material degradation [36, 37]. A lower porosity increases structural strength, whereas a higher porosity enables more tissue ingrowth [8]. The structure of the used scaffolds could have been too dense in our series, although the exact optimal pore size and macroporosity are difficult to determine [38–40]. An increase in macroporosity of the applied scaffold, however, could enable more bone and vessel ingrowth to provide nutrients to the inside of the defect. In case of an adequate fixation of the defect with surgical implants, a nail or a plate, the decreased mechanical strength of the material is less relevant.

The treatment of large bone defects remains difficult, with short-term successes not always ensuring positive long-term outcomes. The addition of a 3D printed custom PCL/TCP scaffold in a large defect aims to improve the retention of biomaterials and to support bone formation. However, further investigations on the impact of micro motion and the structural design of the scaffolds are needed to ensure optimal scaffold behaviour.

## Data Availability

No datasets were generated or analysed during the current study.
